# Eye-Guided Multimodal Fusion: Toward an Adaptive Learning Framework Using Explainable Artificial Intelligence

**DOI:** 10.3390/s25154575

**Published:** 2025-07-24

**Authors:** Sahar Moradizeyveh, Ambreen Hanif, Sidong Liu, Yuankai Qi, Amin Beheshti, Antonio Di Ieva

**Affiliations:** 1 Computational NeuroSurgery (CNS) Lab, Macquarie Medical School, Faculty of Medicine, Health and Human Sciences, Macquarie University, Sydney 2113, Australia; sidong.liu@mq.edu.au (S.L.); antonio.diieva@mq.edu.au (A.D.I.); 2 Centre for Applied Artificial Intelligence, School of Computing, Faculty of Science and Engineering, Macquarie University, Sydney 2113, Australia; ambreen.hanif@mq.edu.au (A.H.); yuankai.qi@mq.edu.au (Y.Q.); amin.beheshti@mq.edu.au (A.B.)

**Keywords:** eye-gaze tracking, deep learning, explanation, artificial intelligence in health

## Abstract

Interpreting diagnostic imaging and identifying clinically relevant features remain challenging tasks, particularly for novice radiologists who often lack structured guidance and expert feedback. To bridge this gap, we propose an Eye-Gaze Guided Multimodal Fusion framework that leverages expert eye-tracking data to enhance learning and decision-making in medical image interpretation. By integrating chest X-ray (CXR) images with expert fixation maps, our approach captures radiologists’ visual attention patterns and highlights regions of interest (ROIs) critical for accurate diagnosis. The fusion model utilizes a shared backbone architecture to jointly process image and gaze modalities, thereby minimizing the impact of noise in fixation data. We validate the system’s interpretability using Gradient-weighted Class Activation Mapping (Grad-CAM) and assess both classification performance and explanation alignment with expert annotations. Comprehensive evaluations, including robustness under gaze noise and expert clinical review, demonstrate the framework’s effectiveness in improving model reliability and interpretability. This work offers a promising pathway toward intelligent, human-centered AI systems that support both diagnostic accuracy and medical training.

## 1. Introduction

Generally, eye-tracking reflects a person’s attentional behavior by measuring their eye movements. This technology provides precise insights into how attention is allocated, making it especially useful in fields that heavily depend on visual information processing, such as medicine and education [1,2]. In medical imaging, eye-tracking data, characterized by specific metrics such as fixation, reflex, and saccades, can significantly enhance deep learning (DL) models by guiding them to areas of interest, thereby improving the accuracy of abnormality detection. This approach addresses a notable challenge in many medical imaging tasks, where the scarcity of large, annotated datasets limits the model’s ability to learn directly from extensive labeled examples. For instance, analysis of gaze data indicates that radiologists allocate greater attention to specific areas indicative of abnormalities during verbal reporting of findings, compared to silent examination of the image [3]. However, despite their effectiveness, DL models are criticized as “black boxes” due to their lack of transparency, which highlights the need for explainable artificial intelligence (XAI) [4]. Additionally, traditional radiology training relies on extensive practical experience, which can be enhanced by integrating experts’ gaze data to accelerate skill acquisition among novice radiologists [5].

**Figure 1:**
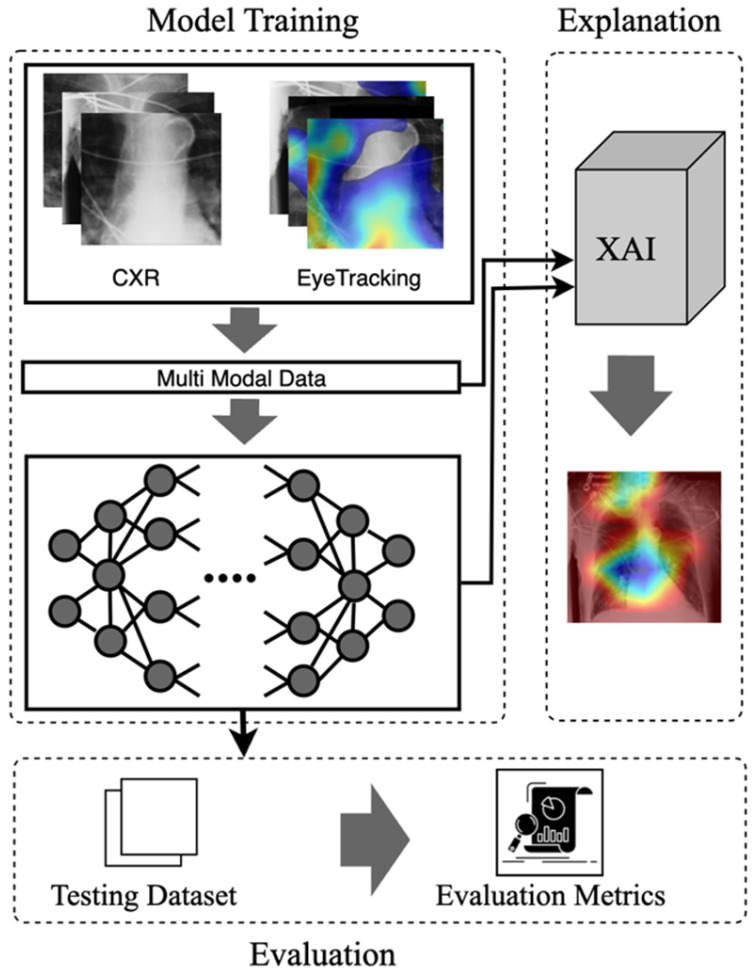
The visual abstract of the eye-guided framework includes the inputs, model, explanation, and evaluation phases.

Recent literature highlights the potential of eye-tracking in medical education, underscoring its ability to differentiate experts from novices through distinct patterns of gaze [6]. Experienced clinicians rapidly form holistic impressions when interpreting medical images, whereas novices typically struggle without substantial practice [7]. With the shift towards online and blended educational environments, integrating eye-tracking data into training programs could bridge this expertise gap. Prior studies indicate that visual characteristics captured through eye movements correlate with cognitive processes involved in clinical reasoning, suggesting a promising approach for knowledge transfer between experts and novices [8]. However, challenges remain, such as handling noisy fixation data and ensuring that gaze-based methodologies align with real-world diagnostic scenarios [9,10]. To address these challenges, this study introduces an Eye-Gaze Guided Multimodal Fusion Framework designed to enhance radiology education and diagnostic accuracy. The overall model and the evaluation steps are illustrated in Figure 1. The key contributions are as follows:

- Eye-Guided Framework: The Multimodal Fusion Framework incorporates attention mechanisms to capture the most crucial aspects of the stimulus. The model with a shared backbone mitigates the impact of noisy fixation data and separates the processing of modalities to improve the performance of automatic abnormality detection in CXRs.
- Explanatory Support for Transparency: We provide post-hoc feature attribution explanations to help radiology trainees better understand lesion classification in chest X-rays.
- Evaluation of the Approach. We evaluate our approach and demonstrate robust performance under noisy conditions, showing resilience to misaligned fixation maps. We further assess the interpretability of the model by utilizing Grad-CAM, ensuring that the generated visual explanations align with expert-annotated ROIs. This alignment enhances the clinical reliability of the model’s predictions.

## 2. Related Work

### 2.1. Eye-Gaze Tracking in Radiology

Eye-tracking technology has been extensively explored for over a decade to study human visual perception, yielding numerous methods for tracking eye movements in various applications. Typically, eye tracking is applied in two main areas: interactive and diagnostic applications [11]. In an interactive context, users’ eye movement data is an input method, whereas in a diagnostic context, it provides insights into their intentions and cognitive processes [1]. While AI advancements have enabled medical deep learning to utilize prior knowledge in diagnostic tools, early studies indicate that eye-tracking technology can enhance clinician interaction and improve AI systems [12,13]. Eye tracking studies in stimulus analysis primarily involve exploring where and how medical professionals examine different modalities of images. Eye movement parameters, such as fixation (the process where the eye remains stationary on a specific point to gather and process visual information), saccades (the rapid eye movements that shift the focus from one point to another quickly), and scanpath (sequence of fixation and saccadic that an individual follows while observing a visual scene), present valuable insights into diagnostic processes and expert decision-making relevant to training [14]. Eye-tracking data influences image interpretation, analysis, and diagnosis. Many studies have discussed utilizing eye-gaze data across diverse applications, including (i) Classification: replicating different search strategies and exploring their potential to improve model performance and interpretability across various modalities [15,16,17]. (ii) Data annotation: to highlight the expert’s search patterns while labeling medical data, including images, text, and genetic information [18,19]. (iii) Object Detection and Segmentation: providing a unique form of supervision format for training DL/ML-based approaches and identifying the precise location of the object [20]. (iv) Workload and Skill: focuses on understanding human factors in medical image interpretation and diagnosis, including the effects of fatigue and skill level on diagnostic accuracy [21].

### 2.2. Multimodal in Medical Data

Multimodal learning in medical applications accelerated in the mid-2010s when integration of various clinical data with multiple types of radiological imaging (e.g., CT scans and MRI) was shown to improve diagnostic precision. Early studies by Li et al. [22] exemplified this paradigm shift, utilizing multiple data sources to provide a more comprehensive analysis. More recently, various medical imaging applications have emerged to integrate eye-gaze tracking data into stimulus analysis. With an increasing focus on multimodal deep learning models, the incorporation of eye-gaze tracking enables a deeper understanding of human search patterns, thereby enhancing the model’s ability to analyze visual information effectively [23]. Drew [24] noted that experts rapidly identify potential lesions, utilize a wider functional field of vision, and draw on more conceptual knowledge than novices when detecting abnormalities. Ma et al. [25] utilized radiologists’ visual attention maps to guide the model in concentrating on task-related objects or features rather than taking harmful shortcuts.

Furthermore, Wang et al. [12] indicate that radiologists’ gaze patterns are strongly linked to diagnostic accuracy in the detection of mammogram lesions. They propose an attention-aware augmentation method that consistently improves focusing contrast and highlights the importance of where radiologists focus, thereby reducing errors in the evaluation phase. Hsieh et al. [26] present a parallel framework that processes chest X-ray images and expert fixation masks through Convolutional Neural Network (CNN) models. This multimodal approach predicts abnormality classes, refines bounding boxes, and optionally generates binary masks for more precise lesion localization. Although analyzing the CXR images and heatmap can cause noise in the model, we discuss in Section 3 the Proposed Model that our approach can mitigate this issue.

### 2.3. Explainable Artificial Intelligence

Several studies have discussed Explainable Artificial Intelligence (XAI) approaches and presented general frameworks for XAI [27,28,29,30,31]. This section discusses studies that develop XAI methods using multimodal data that incorporate eye-tracking information. Currently, only a limited number of studies within the context of multimodal learning have explored explainable systems, despite the recognized significance of XAI systems. In medical imaging, various explanation approaches are visual-based explanations [32]. The core idea of these approaches is to present the information maintained through the model to analyze image regions that contribute most to its predictions. Generally, these methods present attribution maps, which can be used to diagnose a supportive and transparent system. These saliency-based explanations can be categorized into perturbation-based, activation-based, and backpropagation-based techniques [33].

Perturbation-Based Techniques: These methods assess the importance of each input by modifying the image and observing the effect on the model’s output. Perturbation techniques can be applied broadly to classification and regression tasks if the distance between model outputs can be computed. These methods focus on understanding how changes to input features affect the neural activity and predictions of the model.

Activation-Based Techniques: These techniques leverage the feature maps generated in the last layer of a CNN network to explain the model’s predictions. By weighting each feature map and summing them, these methods create class activation maps (CAMs), which highlight areas of the image corresponding to the predicted class. The final activation map is upscaled to the original image’s size, often resulting in a coarse resolution. CAMs help localize regions of interest by detecting neuronal activity associated with specific classes of neurons. Shallow CAMs capture finer details, while deeper CAMs identify broader areas of objects.

Backpropagation-Based Techniques: These methods propagate the output gradients backward to the input image, creating a high-resolution saliency map that highlights which pixels most influence the model’s prediction. Gradients indicate the extent to which each pixel influences the final decision, either increasing or decreasing the predicted class score. These techniques focus on the impact of individual pixels and how changes affect neuron activity.

## 3. The Proposed Model

**Figure 2:**
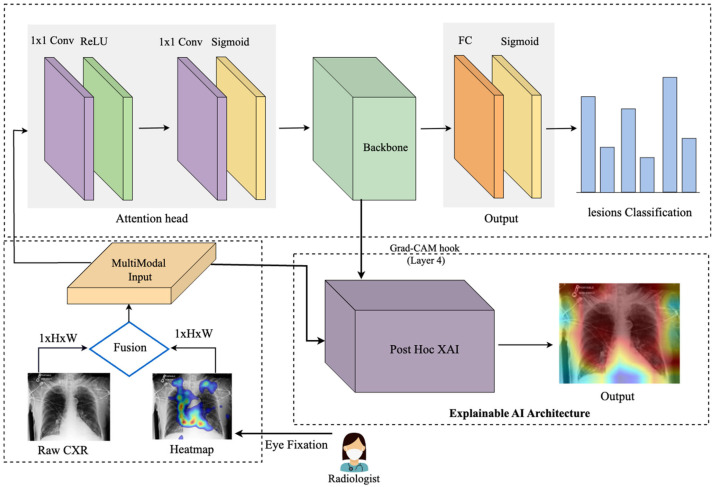
An eye-guided architecture for multimodal learning to enhance abnormality detection in chest X-rays by incorporating radiologists’ eye-tracking data as additional input. The framework combines raw chest X-rays and a heatmap derived from eye fixation data (where blue denotes low fixation density and red denotes the highest concentration of fixations), creating a multimodal input that is processed through an attention head to guide the model’s focus. The unified backbone processes this guided input, followed by a fully connected layer and a sigmoid activation for multilabel lesion classification. The Explainable AI module, based on Gradient-weighted Class Activation Mapping (Grad-CAM), provides post hoc visual explanations. Specifically, after model training, Grad-CAM highlights the critical regions within the chest X-rays that influenced the model’s predictions, enhancing interpretability and transparency for clinical users.

This work introduces an eye-guided multimodal fusion with shared parameters to facilitate interactive exploration and visualization techniques. Our system enables us to use the collected knowledge base on the expert’s fixation map to quickly navigate to reading stimuli while receiving feedback. As shown in Figure 2, the framework of our proposed method consists of three main components. Firstly, after preprocessing raw CXR and the expert’s fixation heatmap, the fusion of feature maps from both modalities enables the model to learn correlations between the eye-tracking heatmaps’ spatial patterns and the X-ray images’ visual features. Secondly, this framework features a unified core between the CXR and fixation map to mitigate the impact of noisy fixation data and prevent the need for separate processing of modalities, which assumes that both sources contribute independently to improved abnormality detection in CXRs. Finally, we evaluate the model’s explanation interpretability through Grad-CAM, its usability, and its impact on radiologist decision-making.

### 3.1. Multimodal Input Data

The input image used in this framework consists of the CXR images. After collecting the raw eye gaze, we generate a related expert’s fixation heatmap using the coordinated points. The eye-tracking heatmap data captures the CXR regions that received the most attention from the radiologist. Then, we applied preprocessing, such as resizing, cropping, and augmentation, including a Gaussian blur to provide a slight blur and avoid overfitting, and color jitter as a brightness and contrast augmentation.

Input data represented as:

$$I_{CXR}\;\in \;R^{W\;\times \;H\;}$$

$$H_{Eye}\in R^{W\times H}$$

where W and H represent the width and height of the image, the single channel corresponds to the grayscale chest X-ray data, and the three channels correspond to the RGB in heatmap overlay data.

### 3.2. Mode

Multimodal Data Processing: The model initially constructs a multichannel input by stacking the chest X-ray images and eye-tracking data along the channel dimension. This early fusion of feature maps from both modalities enables the model to capture and learn correlations between the spatial patterns of the eye-tracking heatmaps and the visual features of the X-ray images. This fusion allows the model to process both data types simultaneously.

(1) $$I_{Multi}=\mathrm{Concat}\left(I_{CXR},H_{Eye}\right)\in R^{W\times H}$$

Attention Heads: The attention mechanism enables the model to focus on the most relevant regions of the input, acting as a filter and aligning the network with human decision-making, potentially improving performance. It processes the input, applies attention scores, and outputs the weighted feature maps. The attention head consists of two 1 × 1 convolutional layer. The first convolution reduces the feature map dimensions, applying ReLU for non-linearity, and the second convolution computes attention scores, followed by a sigmoid activation to normalize the attention weights to the [0, 1] range, and finally, consider adding a dropout layer after attention-scores, as this can help prevent overfitting. The output is element-wise multiplied by the input to generate attended features. The attention head applies the following operations:

(2) $$F_{\mathrm{conv}1}=\mathrm{ReLU}\left(W_{1}\;*\;F_{\mathrm{input}}+b_{1}\right)$$

where W_1_ and b_1_ are the weights and biases of the first convolutional layer, and ∗ denotes convolution. The second layer computes attention scores, followed by a sigmoid activation to constrain the attention weights to the range [0, 1]:

(3) $$A_{\mathrm{weights}}=\sigma \left(W_{2}\;*\;F_{\mathrm{conv}1}+b_{2}\right)$$

W_2_ and b_2_ are the weights and biases of the second convolutional layer, representing the sigmoid function. Finally, the attended features are computed by element-wise multiplying the input feature maps with the attention weights. The attention weights A (weights) are element-wise multiplied by the original input feature map F (input) to produce the attended feature map:

(4) $$F_{\mathrm{attended}}=F_{\mathrm{input}}⊙A_{\mathrm{weights}}$$

Unified Backbone: We use a convolutional neural network model as a shared backbone network. The shared backbone module processes the attended features extracted from the attention head and outputs deep, high-level feature maps. These feature maps are then passed to the fully connected layer, which predicts six labels in the classification task. Since each image can have multiple labels, the fully connected (FC) layer produces independent predictions for each of the six labels. The attended input $F_{\mathrm{attention}}$ from the attention head is passed through the ResNet-50 backbone, which creates the high-level feature representation, denoted as:

(5) $$F_{\mathrm{backbone}}=\mathrm{BackboneModel}\left(F_{\mathrm{attention}}\right)$$

where $F_{\mathrm{attention}}\in R^{W\times H\times 4}\;$ is the multimodal input (combining the X-ray and eye-tracking heatmap) and the backbone output, $F_{\mathrm{backbone}}\in R^{2048},$ which contains the high-level extracted features.

Fully Connected Layer: The final fully connected layer (FC) of the CNN backbone maps the high-dimensional feature vector $F_{\mathrm{backbone}}$ into six output logits for multilabel classification:

(6) $$y_{\mathrm{pred}}=W_{\mathrm{fc}}\cdot F_{\mathrm{backbone}}+b_{\mathrm{fc}}$$

where $\sigma \left(\cdot \right)$ is the sigmoid function that produces a probability for each class. The network then assigns a label to each class based on a predefined threshold (e.g., 0.5). Each of the six labels in the multilabel classification task is converted into a probability using the sigmoid activation function [34]:

(7) $$\sigma \left(x\right)=\frac{1}{1+e^{-x}}\;,$$

where

(8) $${P\;}_{class}=\sigma \left(y_{\mathrm{pred}}\right)$$

### 3.3. Explanation

In the first step, the heatmap generation process involves capturing activations and gradients from the convolutional layer of the backbone model (here, we used the fourth layer). In this study, we employ Grad-CAM as the Post-Hoc XAI component of our framework. Specifically, Grad-CAM provides model interpretability after training by visually highlighting the image regions that most influenced the model’s decisions. A forward hook is registered on the target layer to store the output activations and gradients. During the backward pass, gradients are accumulated with respect to a specific target class, enabling focused visualization. The captured gradients $\frac{\partial y^{c}}{\partial A_{ij}^{k}}$ are pooled across spatial dimensions by averaging:

(9) $$\alpha_{k}^{c}=\frac{1}{Z}\sum_{i}\sum_{j}\frac{\partial y^{c}}{\partial A_{ij}^{k}}$$

Here $y^{c}$ is the score for the target class $c,A_{ij}^{k},\;which$ refers to the activation at spatial location $\left(i,j\right)$ for the k−th  feature map, and Z is the total number of spatial locations $\left(i.e.,i\times j\right).$ These pooled gradients $\alpha_{k}^{c}$ are used to weight the corresponding activations.

The weighted activations are averaged across channels to generate the initial heatmap using the Grad-CAM method [35].

(10) $$L_{\mathrm{Grad}\text{-}\mathrm{CAM}}^{c}=\mathrm{ReLU}\left(\sum_{k}\alpha_{k}^{c}A^{k}\right)$$

A ReLU operation ensures that only positive values are retained, highlighting the relevant areas of interest. In the next step, we normalized and vertically flipped the image to correct orientation issues and match the original image dimensions, thereby building consistent scaling for the heatmaps. Finally, we converted it to an RGB format using a colormap for visual clarity and generated an interpretable visual output.

## 4. Experiments and Results

In this section, we discuss the experimental phase of our research and provide an overview of the key components that contribute to the study. We conclude with a comprehensive discussion that synthesizes our findings and offers a detailed interpretation of the results, aiming to provide a nuanced understanding of our research outcomes.

### 4.1. Dataset

Our experimental validation utilized the REFLACX dataset (Reports and Eye-tracking Data for Localization of Abnormalities in CXR) [36], which is derived from the MIMIC-CXR dataset [37]. Eye-tracking data for this dataset was collected using an Eyelink 1000 Plus system (SR Research, Oakville, ON, Canada) at a 1000 Hz resolution. The system tracked the radiologists’ pupil positions, and the fixation data were synchronized with timestamps from the dictations. Five radiologists provided manual labels for abnormalities and drew ellipses around localized findings over three phases. In the initial testing phase, radiologists reviewed a shared set of 59 CXRs. In the refinement phase, instructions were provided to standardize the labeling process, improving clarity and reliability. It also sets the stage for larger-scale data collection. Eventually, in the primary data collection phase, each radiologist independently reviewed around 500 CXRs. The final dataset was created, which contained eye-tracking data, transcription data, and manual annotations.

**Figure 3:**
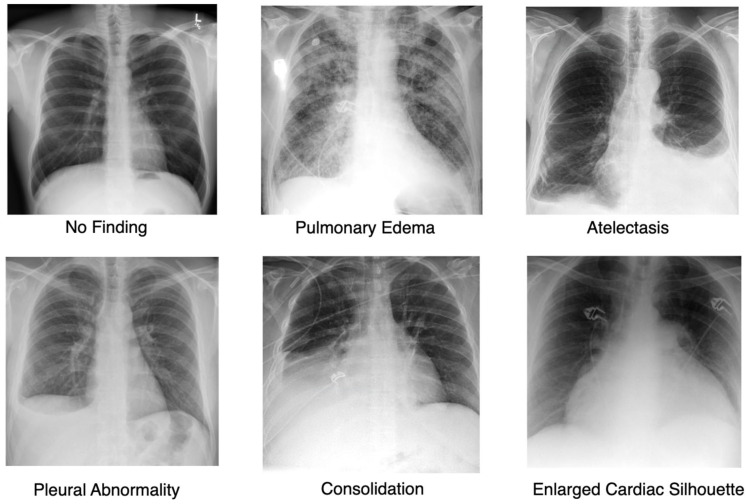
Representative Chest X-ray Images for Different Pulmonary Conditions.

**Figure 4:**
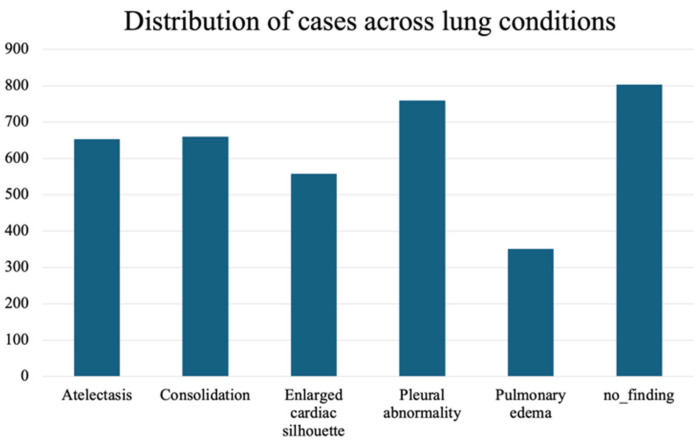
Distribution of the selected most frequently occurring lesion cases across different lung conditions.

To validate the approach, we focus on the most frequently occurring lesions in this dataset, including pleural abnormalities, consolidation, pulmonary edema, enlarged cardiac silhouette, atelectasis, and X-rays showing no specific disease findings. The pleural abnormality represents an abnormal condition of the pleura, the thin tissue that lines the chest cavity and surrounds the lungs. Pulmonary edema is a condition characterized by the accumulation of excess fluid in the lungs. The fluid accumulates in the air sacs, making it difficult to breathe. An enlarged cardiac silhouette is evident when the heart appears more prominent than usual on imaging tests, such as a chest X-ray. Atelectasis is a partial or complete collapse of one or both lungs, which can lead to shortness of breath and difficulty breathing. Finally, consolidation is filling alveolar airspaces with fluid (exudate, transudate, or blood), inflammatory cells, tissue, or other materials. Figure 3 represents CXRs for selected pulmonary conditions analyzed in our research, and Figure 4 displays the distribution of these selected lesion cases across different lung conditions. These visual examples, shown in Figure 3, illustrate the typical radiological appearances, while Figure 4 quantifies their occurrence across the dataset. All data were sourced from the REFLACX dataset, which we describe in detail in Section 4.1.

### 4.2. Implementation Details

**Figure 5:**
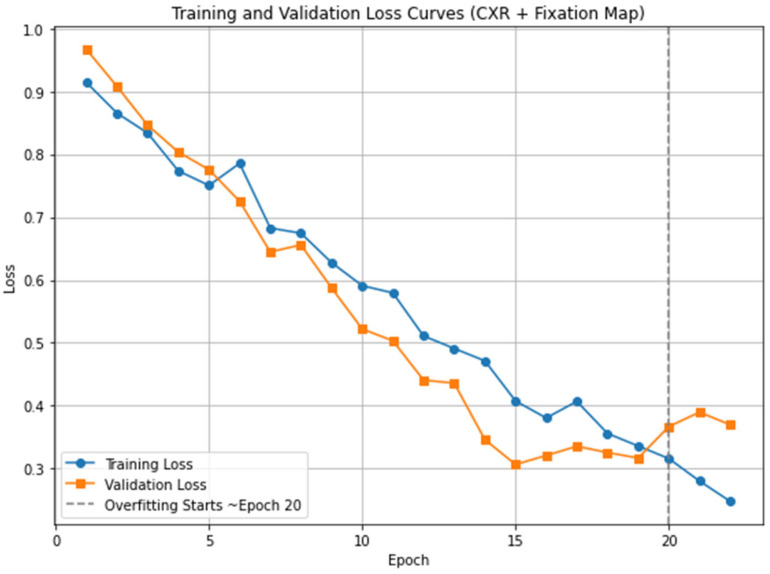
Training and validation loss curves for the proposed model (CXR + Fixation Map). The vertical dashed line marks the point where overfitting begins, approximately after epoch 20.

In this study, we tuned our model for 20 epochs. This value was selected based on an analysis of the training and validation loss curves, which showed that after 20 epochs, the model began to overfit, as indicated by an increasing validation loss while the training loss continued to decline. To prevent overfitting and ensure optimal generalization, we stopped training at this point. The loss curves for the proposed Eye-Guided Multimodal Fusion model are illustrated in Figure 5. We employed an initial learning rate of 5 × 10^−5^ and a weight decay of 1 × 10^−3^. The Adam optimizer was utilized with a batch size of 32 for optimization. The images were cropped and resized to 224 × 224 pixels, aligning with the input requirements of the ResNet-50 architecture. The experiments leveraged open-source model weights pre-trained on ImageNet, which were then fine-tuned on the REFLACX datasets. The experimental setup involved computational resources from a local environment, where we trained and tested the model using version 2.4.1 of the PyTorch framework. The training was conducted on an internal server with an NVIDIA RTX 6000 GPU and an Intel(R) Xeon(R) w7-3465X CPU. Initially, we divided the dataset into training, validation, and test sets using an 80/10/10 split, consistent with our division strategy. A random seed of 42 was used to guarantee reproducibility of the results.

### 4.3. Evaluation Results

This section presents the evaluations performed and the corresponding results. The Eye-Gaze Guided Fusion System was evaluated through a two-phase assessment, comprising both model performance analysis and interpretability assessment using Grad-CAM. Clinical experts subsequently reviewed these evaluations to validate the system’s clinical relevance.

Multimodal Fusion Performance: The effectiveness of the Eye-Guided Fusion System was evaluated by comparing its performance across two configurations: (1) using only CXR images, and (2) combining CXR images with fixation maps as input. The comparison focused on the system’s ability to detect abnormalities, as measured by key metrics including accuracy, precision, recall, and F1-score. To assess the system’s practical reliability, robustness evaluations were conducted under varying conditions by introducing artificial noise into the fixation data, simulating potential misalignments or noise typical in real-world applications. This analysis aimed to determine the system’s capacity to maintain performance despite noisy or degraded inputs, reflecting its robustness for clinical deployment.

Ablation Study on Modality Contribution: The system’s performance, integrating CXR and fixation map modalities, was assessed through an ablation study using ResNet-18 [38] and ResNet-50 [39]. This study compared the effectiveness of various input combinations. The results, summarized in Table 1, indicate that utilizing both modalities in the average eye-guided framework improved metrics. This table presents the results, including per-class metrics for all six findings, as well as the average accuracy scores across all classes and metrics.

**Figure 6:**
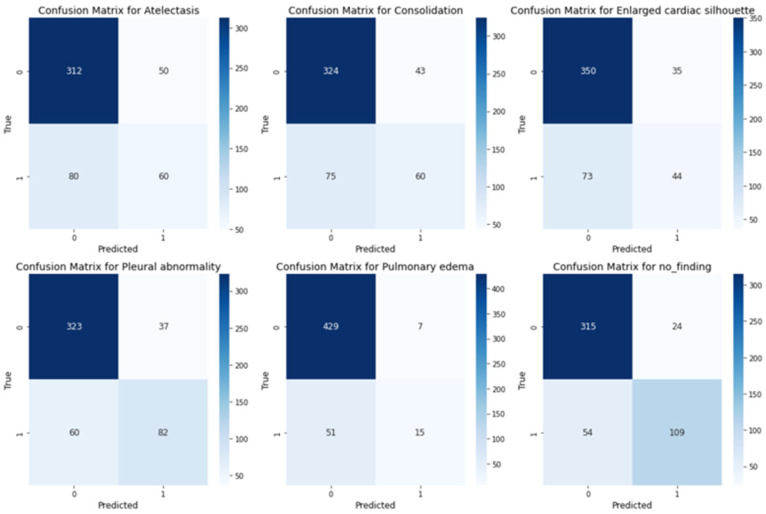
Confusion matrices for multilabel classification across the medical conditions.

As shown in the confusion matrices in Figure 6, for most categories, such as Pulmonary Edema, Atelectasis, Consolidation, and Pleural Abnormality, the number of true positives is substantially higher than the number of false positives and false negatives. Additionally, categories like Pulmonary Edema and Enlarged Cardiac Silhouette exhibit few false positives, indicating the model’s precision and ability to avoid overpredictions. Furthermore, the low false negative rate in these categories suggests that the model effectively captures true positives.

**Figure 7:**
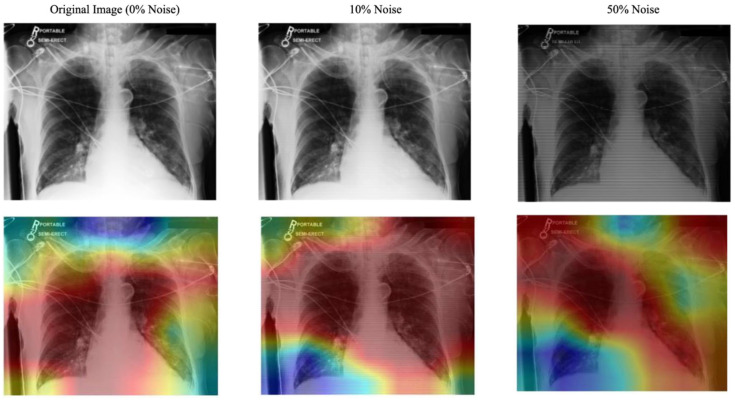
Effect of Striped Noise on Fixation Map with Varying Noise Levels and Stripe Thickness. The blue denotes low fixation density and red denotes the highest concentration of fixations.

Noise Robustness: Artificial noise was introduced into both models to measure how well the system and the explanation component handle noisy or misaligned data. In real-world settings, eye-tracking devices may not perfectly align with the displayed content or the target region of interest (e.g., an X-ray). Minor calibration errors, head movements, or device drift can cause slight shifts in fixation points. In practical applications, eye-tracking data often encounters challenges such as slight misalignment due to device calibration errors, minor head movements, or sensor drift. In this context, we used striped line noise at 10% and 50% levels. A sample of this alignment is shown in Figure 7. This alignment helped test the model’s robustness to minor misalignments that may occur in practice. As shown in Table 2, the eye-guided explanation system demonstrated better noise tolerance results than models without shared parameters, maintaining AUC values at increasing noise levels during testing.

Grad-CAM Explanation Evaluation: In this section, we will present the Intersection Over Union (IoU) performance evaluation of the explanation generated using the Grad-CAM.

Explanation Quality: The Grad-CAM heatmaps were compared to expert-labeled ROI and fixation maps. The overlap between the Grad-CAM activations and experts’ ROIs was measured in multiple classes using the mean Intersection over Union (mIoU) metric, which is defined as:

(11) $$\mathrm{mIoU}=\frac{1}{N}\sum_{i=1}^{N}{\mathrm{IoU}}_{i}\;$$

where N is the total number of classes, and ${\mathrm{IoU}}_{i}$ is the Intersection over Union for class i. The results are illustrated in Table 3.

A radiologist with over 12 years of experience in interpreting radiological imaging evaluated the interpretability and clinical relevance of the Grad-CAM explanations. Evaluations were conducted using a 5-point Likert scale, focusing on clarity, clinical applicability, and diagnostic utility. The radiologist’s repeated assessments across various imaging tasks provided quantitative ratings and in-depth qualitative feedback, highlighting the system’s strengths and limitations in real-world settings. As the radiologist’s familiarity with the eye-guided system increased, there was a marked enhancement in workflow efficiency, evidenced by improvements in diagnostic speed, accuracy, and confidence. Notably, the analysis revealed a progressive alignment between the radiologist’s preferred regions of interest and those identified by the system, further validating its clinical utility. The results of this evaluation are summarized in Table 4.

## 5. Discussion

The experimental findings demonstrate the effectiveness of our Eye-Gaze Guided Multimodal Fusion framework in enhancing abnormality detection in chest X-rays by incorporating radiologists’ eye-tracking data. Compared to the baseline models that rely solely on image data, the inclusion of expert fixation maps significantly improved classification metrics across all six targeted conditions. This suggests that gaze-based guidance provides valuable prior knowledge about salient diagnostic regions, enabling the model to focus its attention more effectively. One significant finding was the framework’s robustness to noise in the gaze data. When artificial noise was introduced to the fixation maps, the system maintained high AUC scores, outperforming the baseline models that did not share parameters. This highlights the value of using a shared backbone architecture, which helps mitigate the influence of noisy or slightly misaligned input data. This property is critical for real-world clinical deployment, where perfect eye-tracking alignment cannot always be guaranteed. Furthermore, the Grad-CAM explanation module demonstrated strong alignment between the model’s visual focus and expert-annotated ROIs. Both quantitative evaluations using IoU and qualitative feedback from experienced radiologists confirmed that the model’s attention maps were consistent with expert diagnostic reasoning. This not only supports the reliability of the system but also enhances its interpretability, a key requirement for the adoption of AI tools in clinical practice. Compared to prior studies in multimodal medical AI, our approach provides a unified and interpretable architecture that leverages human gaze as a form of weak supervision. This approach avoids the need for extensive pixel-level annotation, reflecting how clinicians naturally interpret images. However, the model still has limitations. The fixation data used in training are limited to a small number of radiologists, and only one expert reviews each image.

## 6. Conclusions

In this study, we introduced an Eye-Gaze Guided Multimodal Fusion framework that incorporates radiologists’ eye-tracking data to improve the classification of abnormalities in chest X-rays. By integrating visual attention with image data, the model leverages expert domain knowledge and achieves improved accuracy across various conditions. The proposed approach enhances the interpretability of model predictions through post hoc visual explanations, supporting trust and usability in clinical workflows. This work highlights the potential of human-centered AI systems to support diagnostic imaging and radiology education, particularly in contexts where labeled data or training resources are limited.

### 6.1. Potential Impacts

Although the REFLACX dataset, one of the most valuable resources, used five different radiologists for data preparation, each radiologist examined approximately 20% of the CXRs based on their strategy. However, there is still no public dataset where many experts work on the same images. This issue becomes prominent when different radiologists employ varying search strategies, particularly for images with no significant findings. As we realized, this could introduce bias in these cases.

### 6.2. Future Work

In the future, we will continue to optimize the proposed system by exploring multimodal approaches, particularly by integrating clinical reports alongside image and gaze data. This approach could further improve the model’s understanding and interpretation of its predictions. Generating human-like explanations by simulating how an expert might describe their focus while analyzing an image could also help in correcting biases. Meanwhile, developing a visual dashboard that showcases a real-time, interactive system will illustrate how radiologists can leverage the fusion of eye-tracking and X-ray data to improve diagnosis.

## Figures and Tables

**Table 1:** Ablation study on the efficacy comparison of different modalities. This comparison of per-class and average performance between ResNet18 and ResNet50 models, using chest X-ray (CXR) images alone and in combination with expert fixation maps. Metrics reported include accuracy (ACC), precision, recall, and F1 score for six clinically relevant categories. The average (AVG) across all classes is provided to summarize overall model performance. This table addresses class-level variations and highlights the impact of incorporating gaze data under conditions of class imbalance.

Pulmonary Conditions	CXR Only				CXR + Fixation Map			
	ACC	Precision	Recall	F1	ACC	Precision	Recall	F1
**Model: ResNet18**								
Atelectasis	0.724	0.575	0.534	0.554	0.749	0.527	0.584	0.554
Consolidation	0.739	0.663	0.587	0.623	0.782	0.619	0.609	0.614
Pleural Abnormality	0.812	0.425	0.496	0.458	0.801	0.457	0.496	0.476
Pulmonary Edema	0.757	0.454	0.485	0.469	0.794	0.351	0.485	0.407
No-Finding	0.835	0.400	0.535	0.458	0.806	0.407	0.535	0.462
Enlarged Cardiac Silhouette	0.796	0.630	0.567	0.597	0.826	0.784	0.567	0.658
AVG	0.777	0.525	0.534	0.527	0.790	0.524	0.546	0.529
**Model: ResNet50**								
Atelectasis	0.768	0.467	0.564	0.511	0.769	0.561	0.581	0.571
Consolidation	0.781	0.525	0.525	0.525	0.788	0.579	0.585	0.582
Pleural Abnormality	0.818	0.483	0.522	0.502	0.853	0.551	0.618	0.583
Pulmonary Edema	0.793	0.526	0.542	0.534	0.828	0.687	0.592	0.636
No-Finding	0.834	0.684	0.536	0.601	0.847	0.719	0.643	0.679
Enlarged Cardiac Silhouette	0.816	0.523	0.563	0.542	0.849	0.637	0.617	0.627
AVG	0.801	0.529	0.542	0.535	**0.824**	**0.620**	**0.606**	**0.613**

**Table 2:** Noise robustness of the gaze-guided system.

Noise Level (%)	CXR AUC	CXR+ Fixation Map AUC (No Shared Params)
0	81.41	83.30
10	80.28	81.08
50	74.61	75.86

**Table 3:** Grad-CAM explanation quality (IoU with expert ROI).

Model	IoU Score (Mean ± Std. Dev.)
CXR Only	0.56±0.16
CXR + FixationMap	0.61±0.05

**Table 4:** Expert review of the Grad-CAM explanation.

Criteria	Average Rating (1 to 5)
Interpretability	4.25
Clinical Relevance	4.00

## Data Availability

We utilized the REFLACX dataset, available at Physionet (https://physionet.org/content/reflacx-xray-localization/1.0.0/). Published: 27 September 2021, Version: 1.0.0.

## References

[1] Holmqvist K., Nystr M., Andersson R., Dewhurst R., Jarodzka H., Van de Weijer J. (2011). Eye Tracking: A Comprehensive Guide to Methods and Measures.

[2] O’Brien H.L., Cairns P., Hall M. (2018). A practical approach to measuring user engagement with the refined user engagement scale (ues) and new ues short form. Int. J. Hum.-Comput. Stud..

[3] Moreira C., Nobre I.B., Sousa S.C., Pereira J.M., Jorge J. Improving x-ray diagnostics through eye-tracking and xr. Proceedings of the 2022 IEEE Conference on Virtual Reality and 3D User Interfaces Abstracts and Workshops (VRW).

[4] Chen X., Wang X., Zhang K., Fung K.-M., Thai T.C., Moore K., Mannel R.S., Liu H., Zheng B., Qiu Y. (2022). Recent advances and clinical applications of deep learning in medical image analysis. Med. Image Anal..

[5] Borys K., Schmitt Y.A., Nauta M., Seifert C., Kr N., Friedrich C.M., Nensa F. (2023). Explainable ai in medical imaging: An overview for clinical practitioners–beyond saliency-based xai approaches. Eur. J. Radiol..

[6] Mohajir B.E.E. (2019). Identifying learning style through eye tracking technology in adaptive learning systems. Int. J. Electr. Comput. Eng..

[7] Castner N., Eivazi S., Scheiter K., Kasneci E. (2017). Using Eye Tracking to Evaluate and Develop Innovative Teaching Strategies for Fostering Image Reading Skills of Novices in Medical Training. Eye Tracking Enhanced Learning (ETEL2017).

[8] Kok E.M., Jarodzka H. (2017). Before your very eyes: The value and limitations of eye tracking in medical education. Med. Educ..

[9] Ashraf H., Sodergren M.H., Merali N., Mylonas G., Singh H., Darzi A. (2018). Eye-tracking technology in medical education: A systematic review. Med. Teach..

[10] Newport R.A., Liu S., Di Ieva A. (2024). Analyzing eye paths using fractals. The Fractal Geometry of the Brain.

[11] Duchowski A.T. (2002). A breadth-first survey of eye-tracking applications. Behav. Res. Methods Instrum. Comput..

[12] Wang S., Zhuang Z., Ouyang X., Zhang L., Li Z., Ma C., Liu T., Shen D., Wang Q. (2023). Learning better contrastive view from radiologist’s gaze. arXiv.

[13] Ma C., Jiang H., Chen W., Li Y., Wu Z., Yu X., Liu Z., Guo L., Zhu D., Zhang T. Eye-gaze guided multimodal alignment for medical representation learning. Proceedings of the The Thirty-Eighth Annual Conference on Neural Information Processing Systems.

[14] Moradizeyveh S., Tabassum M., Liu S., Newport R.A., Beheshti A., Di Ieva A. (2024). When eye-tracking meets machine learning: A systematic review on applications in medical image analysis. arXiv.

[15] Zhu H., Salcudean S., Rohling R. Gaze-guided class activation mapping: Leverage human visual attention for network attention in chest x-rays classification. Proceedings of the 15th International Symposium on Visual Information Communication and Interaction.

[16] Ji C., Du C., Zhang Q., Wang S., Ma C., Xie J., Zhou Y., He H., Shen D. (2023). Mammo-net: Integrating gaze supervision and interactive information in multi-view mammogram classification. International Conference on Medical Image Computing and Computer-Assisted Intervention.

[17] Zhu H., Rohling R., Salcudean S. (2022). Jointly boosting saliency prediction and disease classification on chest x-ray images with multi-task unet. Annual Conference on Medical Image Understanding and Analysis.

[18] Teng C., Lee L.H., Lander J., Drukker L., Papageorghiou A.T., Noble J.A. Skill characterisation of sonographer gaze patterns during second trimester clinical fetal ultrasounds using time curves. Proceedings of the 2022 Symposium on Eye Tracking Research and Applications.

[19] Mariam K., Afzal O.M., Hussain W., Javed M.U., Kiyani A., Rajpoot N., Khurram S.A., Khan H.A. (2022). On smart gaze-based annotation of histopathology images for training of deep convolutional neural networks. IEEE J. Biomed. Health Inform..

[20] Stember J.N., Celik H., Gutman D., Swinburne N., Young R., Eskreis-Winkler S., Holodny A., Jambawalikar S., Wood B.J., Chang P.D. (2020). Integrating eye tracking and speech recognition accurately annotates mr brain images for deep learning: Proof of principle. Radiol. Artif. Intell..

[21] Pershin I., Mustafaev T., Ibragimova D., Ibragimov B. (2023). Changes in radiologists’ gaze patterns against lung x-rays with different abnormalities: A randomized experiment. J. Digit. Imaging.

[22] Li W., Jia F., Hu Q. (2015). Automatic segmentation of liver tumor in ct images with deep convolutional neural networks. J. Comput. Commun..

[23] Peng P., Fan W., Shen Y., Liu W., Yang X., Zhang Q., Wei X., Zhou D. (2024). Eye gaze guided cross-modal alignment network for radiology report generation. IEEE J. Biomed. Health Inform..

[24] Drew T., Evans K., Võ M.L.-H., Jacobson F.L., Wolfe J.M. (2013). Informatics in radiology: What can you see in a single glance and how might this guide visual search in medical images?. Radiographics.

[25] Ma C., Zhao L., Chen Y., Wang S., Guo L., Zhang T., Shen D., Jiang X., Liu T. (2023). Eye-gaze-guided vision transformer for rectifying shortcut learning. IEEE Trans. Med. Imaging.

[26] Hsieh C., Luís A., Neves J., Nobre I.B., Sousa S.C., Ouyang C., Jorge J., Moreira C. (2024). Eyexnet: Enhancing abnormality detection and diagnosis via eye-tracking and x-ray fusion. Mach. Learn. Knowl. Extr..

[27] Adadi A., Berrada M. (2018). Peeking Inside the Black-Box: A Survey on Explainable Artificial Intelligence (XAI). IEEE Access.

[28] Carvalho D.V., Pereira E.M., Cardoso J.S. (2019). Machine Learning Interpretability: A Survey on Methods and Metrics. Electronics.

[29] Guidotti R., Monreale A., Ruggieri S., Turini F., Pedreschi D., Giannotti F. (2018). A Survey of Methods For Explaining Black Box Models. ACM Comput. Surv..

[30] Miller T. (2019). Explanation in artificial intelligence: Insights from the social sciences. Artif. Intell..

[31] Tjoa E., Guan C. (2020). A survey on explainable artificial intelligence (xai): Toward medical xai. IEEE Trans. Neural Netw. Learn. Syst..

[32] Van der Velden B.H., Kuijf H.J., Gilhuijs K.G., Viergever M.A. (2022). Explainable artificial intelligence (xai) in deep learning-based medical image analysis. Med. Image Anal..

[33] Gomez T., Mouch H. (2023). Computing and evaluating saliency maps for image classification: A tutorial. J. Electron. Imaging.

[34] Rumelhart D.E., Hinton G.E., Williams R.J. (1986). Learning representations by back-propagating errors. Nature.

[35] Selvaraju R.R., Cogswell M., Das A., Vedantam R., Parikh D., Batra D. Grad-cam: Visual explanations from deep networks via gradient-based localization. Proceedings of the IEEE International Conference on Computer Vision.

[36] Bigolin Lanfredi R., Zhang M., Auffermann W.F., Chan J., Duong P.A.T., Srikumar V., Drew T., Schroeder J.D., Tasdizen T. (2022). REFLACX, a dataset of reports and eye-tracking data for localization of abnormalities in chest x-rays. Sci. Data.

[37] Johnson A.E., Pollard T.J., Greenbaum N.R., Lungren M.P., Deng C.Y., Peng Y., Lu Z., Mark R.G., Berkowitz S.J., Horng S. (2019). MIMIC-CXR-JPG, a large publicly available database of labeled chest radiographs. arXiv.

[38] He K., Zhang X., Ren S., Sun J. Deep residual learning for image recognition. Proceedings of the IEEE Conference on Computer Vision and Pattern Recognition.

[39] Koonce B. (2021). ResNet 50. Convolutional Neural Networks with Swift for Tensorflow: Image Recognition and Dataset Categorization.

